# Optimizing Autologous Fat Grafting: A Systematic Review of Enhancement Strategies and Graft Survival

**DOI:** 10.1093/asj/sjaf242

**Published:** 2025-11-19

**Authors:** Sophie Hasiba-Pappas, Elisa Opriessnig, Sebastian P Nischwitz, Raimund Winter, Patrick Mandal, Martina Carnieletto, Lars-Peter Kamolz, Hanna Luze

## Abstract

Autologous fat grafting has become an important tool in reconstructive and aesthetic surgery, particularly for breast reconstruction and soft tissue augmentation. Despite its promise, long-term outcomes remain unpredictable. To address this, research has increasingly focused on optimizing graft survival by enriching the lipoaspirate with additional substances. A systematic search of PubMed (National Institutes of Health, Bethesda, MD) and Web of Science (Clarivate, Philadelphia, PA) identified studies on enhancement methods of autologous fat grafting in both animal models and clinical settings, published up to February 2025. In vitro trials, case reports, and studies comparing techniques without specific enhancement of the lipoaspirate were excluded. Twenty-seven studies fulfilled the criteria, including 15 animal studies and 12 clinical studies, most of which investigated applications in breast reconstruction and augmentation. The included studies showed substantial heterogeneity in enhancement strategies, case numbers, follow-up duration, and outcome measures. The most frequently examined approaches involved enrichment with stromal vascular fraction (SVF) and/or adipose-derived stem cells (ADSCs). Both methods demonstrated favourable results in many trials, though statistical significance was not always achieved. Other strategies included the use of Vitamin D3, Botulinum toxin A, platelet-rich plasma (PRP), and less frequently studied agents such as N-acetylcysteine and *Salvia miltiorrhiza*. Overall, enrichment of lipoaspirate appears to enhance graft survival, with SVF/ADSC-based methods and PRP emerging as the most promising approaches. Nevertheless, marked variability in protocols and outcome measures hampers comparability between studies. Larger randomized controlled trials employing standardized methodologies are essential to confirm clinical feasibility and provide a basis for evidence-based guidelines.

**Level of Evidence: 3 (Therapeutic)**  
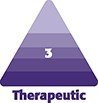

Autologous fat grafting (AFG) has become a transformative option in both aesthetic and reconstructive surgery. First introduced over a century ago for breast augmentation and reconstruction, the technique has since undergone continuous refinement.^1,2^ Its widespread use stems from surgical simplicity, rapid recovery, minimal complications, and immediate results.^1,2^ Despite these advantages, long-term graft viability remains contentious, with fat resorption rates reported between 10% and 90%.^1-3^

Theoretically, strict adherence to established fat grafting principles should ensure consistent, reliable graft take. In practice, however, the challenge of fat resorption often necessitates additional procedures or overcorrection, each introducing distinct limitations. Repeated grafting sessions pose a considerable burden to patients and health care systems and may be restricted by limited donor site availability.^1,2^ Overcorrection, while compensating for anticipated resorption, carries risks of oil cyst formation, fibrotic scarring, and fat necrosis, ultimately exacerbating resorption rates and compromising aesthetic results.^1,2,4^

This dichotomy between the immediate appeal and long-term uncertainty of AFG underscores the need for comprehensive understanding and critical appraisal in both aesthetic and reconstructive contexts. As we delve deeper into the nuances of fat grafting techniques, it becomes evident that elucidating its efficacy, safety, and long-term outcomes is paramount. One of the pressing issues contributing to the uncertainty surrounding graft take and long-term outcome is the marked heterogeneity of enhancement methods and substances investigated in current research. Despite ongoing advancements and numerous studies exploring optimization strategies, no definitive “gold standard” has yet been established.^3^ The aim of this systematic review is to synthesize and critically analyze the available literature on modification techniques for AFG in plastic, reconstructive, and aesthetic surgery, with the goal of identifying effective methods that improve graft take.

## METHODS

This review summarizes the existing evidence published in peer-reviewed journals on the use of lipoaspirate enhancement options in AFG and their impact on the graft take.

### Literature Search

A systematic search of the literature published until February 2025 was conducted on March 4, 2025 using the electronic databases PubMed (National Institutes of Health, Bethesda, MD) and Web of Science (Clarivate, Philadelphia, PA) as well as relevant reference lists. The search strategy included the following terms (variably combined): “autologous fat grafting,” “breast reconstruction,” “lipoaspirates,” “take rate,” and “xenograft.” Additionally, the reference lists of included articles were manually screened for further relevant publications. If the abstract did not determine eligibility, full-text evaluation was performed.

Human studies and in vivo experiments in animal models on the effects of AFG in an aesthetic and/or reconstructive therapeutic setting were determined as the fundamental inclusion criteria. In vitro research was excluded. Full-text evaluation of the publications was performed. All results were independently screened and assessed by 2 researchers to identify any relevant studies for inclusion, and the final set was agreed upon by serial discussion/assessment rounds for any discrepancy in selection. Only articles published in English and German were included.

Studies were eligible for inclusion if they reported the following primary outcome measures: enhancement of lipoaspirates, identification of methods to enhance lipoaspirates, impact on graft take. Studies that solely investigated different harvesting and/or processing techniques were excluded from this systematic review.

PRISMA guidelines were applied to report the systematic review and evidence-based process. The retrieval process reported different a variety of study types. Due to the novel nature of this topic in autologous fat grafting, the guidelines required adaption. To ensure comparability, rigorous critical appraisal was applied to determine the quality of the studies retrieved and ensure inclusion of comparable, valid, and relevant evidence. All results were independently screened and assessed by 2 researchers (S.H.-P. and H.L.) to identify any relevant studies for inclusion, and the final set was agreed upon by serial assessment rounds for any discrepancy in selection.

## RESULTS

A total of 903 studies was retrieved from the systematic search on the database PubMed. One source was obtained from specific searches through other databases (Web of Science; Google Scholar, Alphabet, Inc., Mountain View, CA). After deduplication, addition of automated screening tools and initial screening, 107 PubMed sources remained. Full-text evaluation was performed on 36 papers. Twenty-seven studies investigating lipoaspirate enhancement options for reconstructive and aesthetic purposes were deemed eligible and included in the present systematic review. Fifteen projects focused on in vivo experiments in animal models, 12 studies investigated enhancement methods (Supplemental Table 3, available online at https://doi.org/10.1093/asj/sjaf242) for autologous fat grafting in a clinical setting. Dual independent review of search results and study inclusion was performed by 2 reviewers. The detailed selection process is displayed in Figure 1.

**Figure 1:**
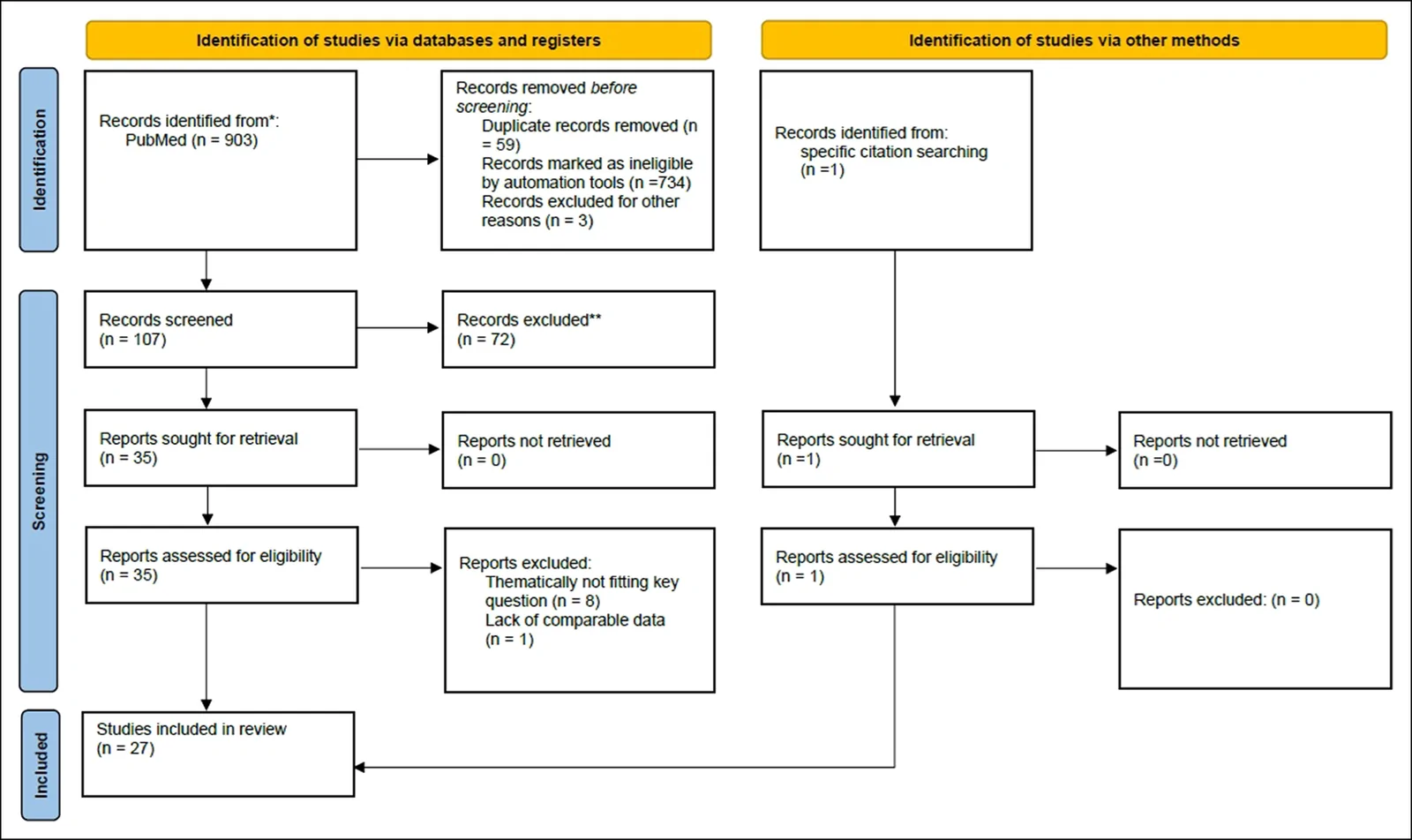
PRISMA 2020 flow diagram for new systematic reviews, which included searches of databases, registers, and other sources. *Consider, if feasible to do so, reporting the number of records identified from each database or register searched (rather than the total number across all databases/registers). **If automation too were used, indicate how many records were excluded by a human and how many were excluded by automation tools. Source: Page MJ et al, 2021;372:n71. doi:10.1136/bmj.n71. This work is licensed under CC BY 4.0. To view a copy of this license https://creativecommons.org/license/by/4.0/.

### Animal Models

This systematic review included 15 studies published between 2015 and 2024. The majority employed rodent models (11 using mice, 1 using rats), while 2 studies used rabbits and 1 evaluated fat grafting in porcine models. Sample sizes ranged from 8 to 60 animals; however, 2 studies did not report animal numbers.^4,5^ All investigations incorporated at least one intervention group and a comparative control. Follow-up periods varied from a minimum of 15 days to a maximum of 2 years.

The most commonly applied outcome measures included graft weight, volume retention (often determined via advanced imaging), oil cyst formation, inflammation, adipocyte viability (cell number, size, or architecture), necrosis, and vascularization. In most studies, fat was harvested from the abdomen or thigh of human donors, whereas only a minority used autologous lipoaspirate from the animal models. Considerable heterogeneity was observed in the enhancement methods applied across studies. Detailed study characteristics are summarized in Supplemental Table 1, available online at https://doi.org/10.1093/asj/sjaf242.

### Adipose-Derived Stem Cells and Stromal Vascular Fraction

Application of adipose-derived stem cells (ADSCs) was reported in 5 studies,^6-10^ stromal vascular fraction (SVF) served as an adjunct in 3 studies.^5,8,9^ In ADSC-enhanced grafts, animals generally demonstrated higher graft retention and reduced oil cyst formation; however, statistical significance was achieved in only one study.^8^ Similarly, SVF-enriched lipoaspirate showed trends toward improved graft take and favourable histological outcomes.

### Vitamin D-Related Substances

One study evaluated the effects of Cholecalciferol (Vitamin D3), administering different doses following lipoaspirate injection.^11^ High-dose cholecalciferol showed superior results compared to low dose and control groups in terms of graft weight, volume, and histological parameters such as vascularization and structure. However, only graft weight and volume reached statistical significance.^11^

Another study investigated Calcitriol, the active form of Vitamin D3 as an enhancement substance for AFG.^4^ Calcitriol was either applied locally or systemically via intraperitoneal injection and compared with the respective control group. Local treatment was administered once, whereas systemic treatment was conducted multiple times. Sustained systemic calcitriol achieved significant greater graft weight and volume than local treatment or controls.^4^

### Other Enhancement Methods

Adenovirus E4+ endothelial cell-assisted lipotransfer (Ad E4+ EC) was investigated in one murine study, with phosphate buffered saline as the control substance. High-dose Ad E4+ EC resulted in significantly greater volume preservation at 90 days after AFG, whereas low-dose treatment maintained superior volume retention at 180 days.^12^

Another group evaluated high-density fat washed with Poloxamer-188 before, a compound with cytoprotective and anti-inflammatory properties. This approach improved stromal vascular fraction (SVF) survival and significantly enhanced vascularization.^13^ Repeated local administration of leptin, delivered via a skin window into the graft, also led to significantly increased vascularization.^14^

Additional adjuncts included human umbilical cord-derived lyophilized stem cells and Thymosin beta4, both of which enhanced graft preservation and angiogenesis.^15,16^

There was one report of low-level laser therapy (LLLT) as a treatment adjunct either alone or in combination with ADSCs.^7^ While both LLLT and ADSC treatment improved graft take, only LLLT alone shows higher VEGF expression.^7^

Traditional Chinese Medicine (TCM) was explored in one study, where systemic administration of *Salvia miltiorrhiza*, more commonly known as “red sage,” for 4 weeks post-AFG significantly increased graft retention and VEGF levels compared with controls.^17^ Finally, porcine acellular dermal matrix mixed with AFG reduced fat liquidation and fat necrosis relative to control groups.^18^

### Clinical Studies

Twelve clinical studies published between 2012 and February of 2025 were included in this review, all focusing on AFG for aesthetic and/or reconstructive breast augmentation. The study populations were predominantly female, with only few studies including both genders. All patients were aged ≥18 years. Follow-up durations ranged from a minimum of 4 weeks to a maximum of 18 months. Reported donor sites included the abdomen, thighs, flanks, hips, and lower legs. Outcome parameters varied across studies, whereby most of them evaluated volume retention (often assessed via 3D scans) and contour restoration. Fewer studies examined postoperative atrophy, cyst formation, and complications. Patient-reported outcomes were inconsistently assessed, with only 1 group applying the Breast-Q questionnaire as the primary measurement tool. Clinical studies are portrayed in Supplemental Table 2, available online at https://doi.org/10.1093/asj/sjaf242.

#### Cell-Assisted Lipotransfer

Cell-assisted lipotransfer (CAL) using either ADSCs or SVF was described in 7 studies.^19,20-25^ The process included ex vivo expansion and isolation of the cells followed by enrichment of the fat graft prior to transfer. Most studies reported improved graft rates, reflected by increased retention rates with exception of Chiu et al,^23^ who observed reduced retention rates and increased complication rate in their retrospective analysis of SVF-enriched AFG. Across studies, patient satisfaction was consistently high. Statistically significant improvements in retention with CAL were specifically demonstrated by Hu et al^24^ and Kolle et al.^21^

#### Platelet-Rich Plasma

Gentile et al^22,26^ conducted 2 clinical trials investigating platelet-rich plasma (PRP) as an adjunct to AFG for breast reconstruction. In the first trial, PRP-enhanced grafts demonstrated superior outcomes, with 69% contour maintenance and 3D volume retention compared to 39% in the standard AFG group.^26^ In a subsequent study, the same authors compared PRP and stromal vascular fraction (SVF) as enrichment strategies. Both interventions improved graft take, with PRP achieving 69% and SVF 63% maintenance, while AFG alone again showed 39%.^22^

#### Botulinum Toxin A

Two recent studies by Wang et al^27^ and Liu et al^28^ investigated Botulinum Toxin A (BoNT-A) as an adjunct in AFG. In both studies, BoNT-A was added to the centrifuged lipoaspirate, resulting in significant higher retention rates compared to standard AFG. Wang et al,^27^ who applied AFG for breast augmentation in patients with Poland syndrome, reported a median number of 3 grafting sessions to achieve satisfactory results, defined as ≥80% fat retention. Patients receiving BoNT-A enhanced grafts also reported significantly higher satisfaction with the aesthetic outcome.^27^

#### Other Enhancement Methods

Pietrusky et al^29^ evaluated the amino acid derivate N-Acetylcysteine (NAC) as an enhancement substance for AFG, adding it to the tumescent solution prior to fat harvesting. Compared with standard liposuction, the NAC group demonstrated lower fat resorption rates. Zheng et al^30^ relied on TCM, administering intravenous *Salvia Miliorrhiza* for 4 weeks after AFG to the breast. While both groups achieved good breast volume, only the treatment group showed improved breast morphology, though without statistical significance.^30^

#### Complications

Overall, the procedures were considered safe. Minor findings included postoperative atrophy, cyst formation, or mild fat necrosis in a limited number of patients.^19,28^ Some studies also reported contour irregularity or revision/additional grafting sessions; however, these were generally reported as outcome parameters or expected sequelae rather than true complications. Importantly, no major adverse events related to AFG or its enhancement methods were reported.

## DISCUSSION

This systematic review synthesized current evidence on strategies to enhance graft take following AFG, drawing from both animal and clinical studies. Within in vivo models, a clear trend toward stem cell research was observed, with 6 out of 15 studies utilizing either SVF or ADSCs as adjuncts to the fat graft.^5-10^ Although SVF contains ADSCs, the 2 terms should not be used interchangeably. ADSCs represent a relatively homogenous population of stem cells that can be applied both autologous and allogenic settings. SVF, on the other hand, is a heterogenous cell mixture that includes ADSCs, endothelial cells (EC), macrophages, and pre-adipocytes and is generally recommended for autologous application only.^31^

Both SVF and ADSCs have been widely studied across various fields such as wound healing, osteoarthritis and, relevant to this review, breast augmentation and reconstruction. Their proposed mechanisms of action extend beyond differentiation into adipose tissue, encompassing paracrine signalling through growth factor release and the promotion of angiogenesis.^32^ While some authors suggest that the heterogeneity of SVF may yield therapeutic benefits, consensus remains lacking.^31,33^ Importantly, adipose tissue itself represents an abundant and accessible source of regenerative cells, yet, without proper isolation, their regenerative potential might not be fully realized in AFG.

CAL seeks to address this limitation by enriching conventional fat grafts with ADSCs or SFV, thereby increasing the survival of transplanted cells and improving long-term graft retention.^34^ This theoretical benefit is partly supported by the trials included in this systematic review: across animal studies, ADSC, or SVV enrichment consistently yielded improved outcome compared to standard AFG, although statistical significance was not uniformly achieved. A critical challenge for CAL remains the attainment of clinically relevant and reproducible cell concentrations. The optimal threshold of ADSCs/SVF supplementation has not been determined, nor has a standardized, universally accepted isolation technique to maximize cell viability.^32^

Consistent with results from in vivo studies, enrichment with ADSCs and SVF generally produced favourable results in clinical studies. Notably, Chiu et al^23^ were the only authors to report inferior results with SVF-enriched AFG. No major protocol discrepancies were identified between this trial and other, making the divergent findings difficult to explain. Nevertheless, the preponderance of evidence supports increased graft uptake and fat retention for months following breast augmentation or reconstruction with cell-enriched AFG.^19-22,24,25^

None of the studies included in this systematic review specifically utilized nanofat as an enrichment component in CAL or other enhancement protocols. Nevertheless, nanofat represents a potential source of stromal vascular fraction and regenerative cells that could theoretically be employed to improve graft integration. Further studies are warranted to determine its safety, feasibility, and effectiveness in this context.

Two studies investigated a fundamentally different approach by enriching AFG with Vitamin D3.^4,11^ Vitamin D3 and its derivates are implicated in modulating cellular stress responses, differentiation, and proliferation,^35^ and they have also demonstrated anti-inflammatory effects on both adipocytes and immune cells.^36^ While the preliminary results Vitamin D3-enhanced AFG were promising, the small number of cases precludes definite conclusions regarding its efficacy. This limitation is even more apparent for other novel approaches such as LLLT or Ad E4+ EC.^7,12^ Despite rigorous methodology, the reliance on single trials makes it difficult to establish the clinical utility of these modalities.

PRP also emerged as a promising adjunct in clinical studies.^22,26^ This aligns with its proposed biological role in enhancing AFG outcomes.^37^ Evidence from osteoarthritis research suggests that the combination of PRP and fat yields superior results compared to either treatment alone,^38^ likely reflecting synergistic effects. Mechanistically, PRP may support graft survivals by modulating local immunogenic responses and improving vascularization through the release of vascular endothelial growth factor and other pro-angiogenic mediators.^39,40^ Although only 2 studies on PRP-enriched lipoaspirate for AFG of the breast were included in this review, both suggest potential benefits, warranting further investigation through larger and more standardized clinical trials.

BoNT-A has also been suggested to increase fat graft retention.^27,28^ Although the underlying mechanisms remain incompletely understood, it has been proposed that BoNT-A fosters a pro-angiogenic environment, thereby improving long-term effects of AFG. Other substances such as NAC or *Salvia Miliorrhiza* have been tested in isolated clinical trials by Pietrusky et al^29^ and Zheng et al,^30^ respectively, and both reposted potentially beneficial effects. However, with only a single study available for each agent, the evidence base remains insufficient for drawing reliable conclusions. Though fewer data are available on methods like PRP and BoNT-A, current research suggests it may also be worthwhile to further investigate their role in improving graft uptake.

Though fewer data are available on methods like PRP and BoNT-A, current research suggests it may also be worthwhile to further investigate their role in improving graft uptake.

As previously mentioned, a consistent trend toward ADSCs and SVF enrichment emerged, with both experimental and clinical data supporting improved graft survival. Further validation through well-designed, randomized controlled studies is essential to determine the clinical efficacy of this approach and to establish standardized protocols. However, the advancement of stem cell–based therapies is hindered by multiple challenges, including the technical complexity of cell isolation procedures, as well as regulatory and ethical considerations surrounding human applications. While data on alternative strategies such as PRP and BoNT-A remain more limited, the encouraging preliminary findings suggest that these adjuncts merit further investigation. Expanded research efforts may ultimately help identify optimal, clinically feasible approaches for enhancing graft retention in AFG.

The primary aim of this systematic review was to highlight and critically discuss current enhancement methods that may influence graft survival after AFG. However, it is important to recognize that multiple additional factors can modulate the efficacy of autologous fat in a therapeutic setting. Donor site selection, harvesting technique, and processing method may each play significant roles in determining graft outcomes.

As demonstrated in the studies included, fat can be harvested from various anatomical sites, including the upper and lower abdomen, flanks, thighs, and lower legs. At present, no clear consensus exists regarding an optimal donor site. Although differences in complication rates and patient satisfaction have been reviewed, the direct impact of donor site on graft take remains underexplored.^41^ Luze et al^42^ compared outcomes in breast reconstruction patients undergoing AFG using fat harvested from the abdomen, upper legs, or lower legs and found no statistically significant correlation between harvest site and graft take. Similarly, Wederfoort et al^41^ reported inconclusive findings with respect to donor site, complications, and patient satisfaction.

Harvesting and processing techniques represent another major variable that may contribute to the often unpredictable outcomes of AFG. A higher yield of viable adipocytes is generally associated with improved graft survival^43^; yet, mechanical stress and other factors during harvesting can compromise cell viability.^44,45^ Technical considerations such as cannula size, use of tumescent solution, and the application of ultrasound- or power-assisted liposuction have all been linked to adipocyte integrity and graft outcomes.^46^ In their comprehensive review, Strong et al^46^ found some evidence suggesting differences in adipocyte viability depending on harvesting method. However, long-term retention rates did not differ significantly across syringe aspiration, suction-assisted liposuction, or ultrasound-assisted liposuction.

Processing methods—including centrifugation, washing, filtration, and manual gauze rolling—have likewise been investigated for their impact on cell survival and graft retention.^46^ Some studies reported higher adipocyte viability with gauze rolling, arguing that centrifugation imposes mechanical strain on cells,^47^ whereas others observed superior outcomes with centrifugation compared to gravity sedimentation.^48^ Consistent with the findings of our review, Strong et al emphasized the high degree of heterogeneity and lack of standardized protocols across studies, which substantially limits comparability and hinders the establishment of best practices.

Taken together, current evidence suggests that while innovative biological adjuncts to AFG—such as ADSCs, SVF, PRP, and others—show considerable promise, the influence of fundamental procedural factors like donor site, harvesting, and processing techniques cannot be overlooked. The interplay between these variables and enhancement methods likely determines the overall efficacy and durability of fat grafting.

At present, the absence of standardized methodologies and the variability across study designs prevent firm conclusions or the formulation of clear clinical guidelines. Future research should prioritize randomized controlled trials with standardized protocols to better define which factors—whether biological adjuncts or technical variables—most critically influence long-term graft survival. Establishing these determinants is a crucial step toward optimizing AFG as a reliable and versatile treatment option in reconstructive and aesthetic surgery.

In addition to the biological and technical aspects discussed, the practical implementation of these enhancement strategies in clinical settings warrants further attention. While CAL using SVF or ADSCs has shown promising outcomes, its routine application remains limited by procedural complexity, time consumption, and substantial costs associated with cell isolation, expansion, and processing equipment.^19,21,31,32,34^ Moreover, the regulatory requirements for manipulating human-derived cells vary considerably across regions, posing additional barriers to standardization and widespread clinical use.^31,32^ Consequently, CAL is currently feasible primarily in specialized centers with access to appropriate laboratory infrastructure and expertise. In contrast, adjuncts such as PRP or BoNT-A appear more accessible, cost-effective, and technically simpler to integrate into standard operative workflows.^22,26-28^ However, further evidence is required to determine their long-term efficacy, reproducibility, and cost–benefit ratio in large-scale practice. Feasibility also depends on surgeon training, institutional logistics, and patient selection, all of which influence procedural success and sustainability. Future studies should therefore address not only biological and clinical outcomes but also the economic, ethical, and operational dimensions of implementation to facilitate the safe and cost-effective translation of these promising techniques into everyday reconstructive and aesthetic surgery.

### Limitations

This research was not without limitations. One very important limitation is the high variability concerning enhancement methods and protocols amongst the included studies. The lack of heterogenous trial protocols inevitably diminishes comparability. Though some enhancement substances and techniques were investigated in multiple studies, including randomized controlled trials (RCTs), some techniques were only reported in one trial, making it difficult to assess their efficacy and determine reliable recommendations on the treatment option. Another factor was high variation in follow-up time after the intervention. This is especially challenging as the aim of AFG enhancement methods is to improve the graft take in the long term. Some of the included studies conducted follow-up periods of 1 month or less; therefore, long-term efficacy can hardly be determined from these reports. A further limitation concerns the accuracy of outcome assessment across the included studies. Imaging modalities such as MRI, CT, or 3D scanning, while valuable for volumetric analysis, may lack precision when applied to small-volume grafts in both animal and human models. Similarly, histological evaluation is limited by sampling error and may not fully represent overall graft viability. These methodological constraints contribute to the variability and limited comparability of reported outcomes across studies.

It should be emphasized that the majority of the studies analyzed in this review were conducted in animal models or using non-human adipose tissue. The limited number of available human trials often suffer from small sample sizes, lack of randomization, and heterogeneous methodologies. As a result, most observations supporting specific enhancement strategies should be interpreted as experimental rather than clinically validated. The current evidence base, while promising, remains largely preclinical and requires further confirmation in well-designed human studies before robust clinical recommendations can be made. Yet, we strongly believe that it is crucial to acknowledge fundamental research as the data provides the groundwork for advanced studies in clinical settings.

## CONCLUSIONS

The potential of AFG has expanded significantly, heralding a new era of innovation and refinement in surgical practice, including the field of breast reconstruction. With this expansion comes a responsibility to continue to advance the status quo. Current research provides enhancement options for lipoaspirates with cell-enrichment techniques and adjunct substances that may greatly improve AFG longevity and efficacy in the future. These advancements hold the potential to contribute to greater graft viability and integration, ultimately, thereby the overall success of the procedure.

However promising, currently available data lack standardized protocols and substantiated recommendations, posing a hardship in the translation into everyday clinical settings. We strongly believe the encouraging results gained from the studies presented in this systematic review need to be further explored under more homogenous and reproducible conditions. Furthermore, more randomized controlled trials are warranted. With ongoing research and further advancements, the future holds promise for further improvements in graft survival, patient satisfaction, and overall surgical outcomes.

## Supplemental Material

This article contains supplemental material located online at https://doi.org/10.1093/asj/sjaf242.

## Supplementary Material

sjaf242_Supplementary_Data
