# Supplementary material for: Optimizing Autologous Fat Grafting: A Systematic Review of Enhancement Strategies and Graft Survival
Source: Aesthet Surg J. 2025 Nov 19;46(11):1246–52. doi: 10.1093/asj/sjaf242 (PMC13668368; doi:10.1093/asj/sjaf242)
Supplement: Supplementary file 1 [file sjaf242_supplementary_data.zip › Supplemental Table 1.docx]

**Supplemental Table 1**. Animal Models Evaluating Lipoaspirate Enhancement Techniques in Autologous Fat Grafting and Their Impact on the Graft Take

| Title and authors | Study Type | Study group | Comparison group(s) | N | Time until follow-up | Technique  (main steps) | Outcome measurement | Main Results  *(* = significant value)* |
| --- | --- | --- | --- | --- | --- | --- | --- | --- |
| Poloxamer 188 washing of lipoaspirate improves fat graft survival: A comparative study in nude mice  (Liu et al., 2024)  Vitamin D3 improves Adipose Stromal Cell Survival and Human fat Graft Retention in Xenograft Model  (Gavrilescu et al., 2024)  Active Vitamin D3 Increases Adipose Graft retention in a Xenograft Model  (Loder et al., 2023)  Improving Fat Transplantation Survival and Vascularization with Adenovirus E4+ Endothelial Cell-Assisted Lipotransfer  (Dong et al., 2023)  The Effect of Human Umbilical Cord-Derived Lyophilized Stem Cells on Fat Graft Viability: An Experimental Study  (Tutak et al., 2022)  Human Adipose Mesenchymal Strom/Stem Cells Improve Fat Transplantation Performance  (Piccinno et al., 2022)  Leptin promotes adipocyte survival in non-vascularized fat grafting via perfusion increase  (Schreiter et al., 2021) | In vivo, Comparative study  In vivo  In vivo  + ex vivo  In vivo  In vivo, Prospective Treatment-Control study  In vivo  In vivo | High density fat washed with P188 (1)  5,000 ng VD3 (1)  Calcitriol local (1), Calcitriol systemic (2)  AdE4+ EC-enriched fat graft  High dose (1) and low dose (2)  FG + MSC-L (1)  FG+ Human autologous AD-MSCs (1)  AD-MSCs + HA (2)  Locally applied leptin (1) | HDF washed with saline  (2)  Control (2), 50 ng calcitriol (3), 50 ng VD3 (4), 500 ng VD3 (5)  Local control (3)  Systemic control (4)  Phosphate buffered Saline fat graft (3)  FG + Saline (2),  Control group w/o FG (3)  FG suspended in HA, no AD-MSCs (3)  Control w Saline  (2) | 16 mice  40 mice  Mice,  N n.m.  28 mice  27 mice  21 mice  44 mice | 48 w  12 w  1, 4, 12 w  90, 180 d  3 m  180 d  15 d | Centrifugation of Lipoaspirate to obtain HDF, washing with saline or P188, cotton concentration, transplantation in mice, explanation and analysis  Collection of lipoaspirate, isolation of ASC,  Injection, treatment with or w/o VD3  Collection of lipoaspirate, in vivo implantation, treatment once local or repeated via intraperitoneal injection  Decantation and processing of human lipoaspirate, adding of AdE4+ EC or PBS, s.c. injection, explanation and analysis  Incision in animal model, direct closure (3) or animal lipoaspirate transplantation on sternum, addition of saline or MSC-L, harvest, analysis  Fat harvest from human abdomen via Coleman technique, Isolation of AD-MSCs, Implantation in mice  S.c. transplantation of FG,  application of leptin or saline 3,7,10 d postop into FG through skin window | Volume retention, histology (SVF survival, Ki-67 positive cells, structure, fibrosis, vascularization)  Graft weight, graft volume, vascularization, oil cysts, adipocyte architecture  Graft weight and volume, Adipocyte viability, fibrosis, vascularization  Volume (3D imaging), necrosis, structure, inflammation, vascularization  Adipose tissue volume, connective tissue volume, adipocyte structure, fibrosis, vessel preservation  Graft weight, resorption rate, lipidic cysts  Vessel density and diameter, adipocyte survival, preadipocyte development | (1) Higher survival rate of SVF, more Ki67, less fibrosis  (1) improved vascularization*  Higher graft weight and volume in (1)*  Enhanced vascularization, intact structure, absent oil cysts in (1)  Graft weight and volume higher with sustained Calcitriol treatment (2)*  (1) highest volume preservation* at 90 d  (2) highest volume preservation at 180 d  Improved vascularization with AdE4+ EC  Higher adipose tissue preservation in (1)  Better preservation of vessels and tissue structure in (1)  Lower resorption rate and reduced lipidic cyst formation in (1) and (2)  Higher vessel density (1)*  Higher expression of preadipocyte markers (1)* |
| Effects of low-level laser therapy and adipose-derived stem cells on the viability of autologous fat grafts: a preliminary study. (Heo *et al.,* 2021) | In vivo | LLLT (2), ADSC (3), LLLT+ADSC (4) | Control (1) | 20 mice | 8 w | Fat harvest from human abdomen, s.c. implantation with respective added technique (LLLT and /or ADSC) | Weight, volume, histology, Western blot findings | Group 2,3,4 higher retention rate and volume  Higher VEGF expression in (2) |
| Porcine Acellular Dermal Matrix Increases Fat Survival Rate after Fat Grafting in Nude Mice. (Zhu *et al.,* 2021) | In vivo | Porcine acellular dermal matrix + AFG mixture (1) | Control, standard AFG (2) | 32 mice | 4 w | Fat harvest from human thigh, washing of fat, addition of PADM (1), injection | Fat weight, liquefaction ratio, histological changes (e.g. fat necrosis) | Less fat liquidation in (1)  Decreased fat necrosis in (1) |
| The Effect of Thymosin beta4 on the Survival of Autologous Fat Grafting: A Preliminary Study. (Qu *et al.,* 2020) | In vivo | Thymosin beta 4 (Tß4) added to AFG:  5ug/mL (1)  10ug/mL (2) | phosphate-buffered saline | 18 Rabbits | 2,4, 12 w | Harves from scapular region of animals, Coleman technique applied, mixing of fat with Tß4 | Volume and weight retention, adipose tissue integrity, adipocyte viability, angiogenesis | better volume, weight retention, tissue integrity, adipocyte viability, angiogenesis and microcirculation in (1) and (2) |
| Cell Enriched Fat Grafting Improves Graft Retention in a Porcine Model.  (Rasmussen *et al.,* 2019) | Dose-response study | ADSC (1) or SVF (2) enriched AFG | Control (3), non-enriched | 13 pigs | 120 days | Harvest of lipoaspirate from pigs, isolation and culture of ADSC or SVF, addition to AFG | Retention rate (MRI), morphological results | Higher graft retention in (1)* and (2)* No sign. different morphology among groups. |
| Endothelial Differentiated Adipose-Derived Stem-Cells Improvement of Survival and Neovascularization in Fat Transplantation. (Harris *et al.,* 2019) | In vivo | AFG enriched with SVF (1), EC/ACS (2) | AFG alone (3) | 8 Rats | 8 w | 3 AFG mixtures implanted into each rat at different location | Survival weight, histological examination, Neo-vascularization | higher survival rate, morphological integrity, less inflammation and fibrosis increased number of vessels in (1) and (2) |
| Positive Effect of Incubated Adipose Derived Mesenchymal Stem Cells on Microfat Graft Survival.  (Choi *et al.,* 2018) | In vivo, immunocompromised model | Incubated ASC + microfat graft (1) | Microfat naïve (2) | 60 mice | 8,16 w | Harvest from human abdomen, ASCs isolated from tissue | Volume, weight, histopathological and immunologic staining | higher fat volume in (1), fewer cysts and fibrosis, higher levels of adipocytes, higher number of capillary vessels in (1) |
| Fate of Free Fat Grafts with or without Adipogenic Adjuncts to Enhances Graft Outcomes. (Tang *et al.,* 2018) | In vivo, immunocompromised model | Added extracellular matrix (1), added SVF (2) | Free fat graft (3) | Mice,  N n.m. | 6-12 w | Lipoaspirate harvest from female patient, placed into scalps of mice | Survival and retention of fat grafts | improved proliferation of murine fat in (1) and (2) |
| Traditional Chinese Medicine: Salvia miltiorrhiza Enhances Survival Rate of Autologous Adipose Tissue Transplantation in Rabbit Model. (Yu *et al.,* 2015) | In vivo Model | e ADSM+AFG (1) | Control AFG (2) | 13 rabbits | 12 w | FG harvested from rabbit scapular, AFG, intraperitoneal SM for 4 w | Fat survival, VEGF levels  Adipocyte survival | Higher graft retention and VEGF levels in (1)*  Higher adipocyte survival |

Abbreviations: N: number of participants, P188:Poloxamer 188, HDF:High density fat, w:weeks, SVF:Stromal vascular fraction, VD3:Vitamin D3, ADSC:Adipose derived stem cell, w/o:withour, n.m.:Not mentioned, m:months, s.c.:subcutaneous, AdE4+:Adenovirus E4+, EC: endothelial cell, d:days, PBS: Phosphate buffered saline, (A)FG:(Autolgous) fat grafting, (AD)MSC: (Adipose-derived) Mesenchymal stem cells, HA:Hyaluronic acid, LLLT:low-level laser therapy, PADM: Porcine acellular dermal matrix, SM: Salvia miltiorrhiza, VEGF: Vascular endothelial growth factor
