# Supplementary material for: Optimizing Autologous Fat Grafting: A Systematic Review of Enhancement Strategies and Graft Survival
Source: Aesthet Surg J. 2025 Nov 19;46(11):1246–52. doi: 10.1093/asj/sjaf242 (PMC13668368; doi:10.1093/asj/sjaf242)
Supplement: Supplementary file 1 [file sjaf242_supplementary_data.zip › Supplemental Table 2.docx]

**Supplemental Table 2**. Clinical Studies Evaluating the Lipoaspirate Enhancement Options in Autologous Fat Grafting and Their Impact on the Graft Take

| Title and authors | Study Type | Objective | Study / control groups | N, Baseline characteristics | Time (until final follow-up) | Technique | Outcome measurement | Results  *(*= statistically value)* |
| --- | --- | --- | --- | --- | --- | --- | --- | --- |
| Autologous Fat Graft Combined with Botulinum Toxin Injection for Breast Augmentation in Poland Syndrome  (Wang et al., 2025)  Improving the Retention Rate of Fat Grafting by Botulinum Toxin A: A randomized, self-controlled Clinical trial  (Liu et al., 2024)  Clinical Efficacy Analysis of Augmentation Mammoplasty with Cell-Assisted Autologous Fat Transplantation  (Hu et al., 2022)  The Impact of N-Acetylcysteine on Autologous Fat Graft: First-in-Human Pilot Study.  (Pietruski *et al.,* 2021) | Prospective comparative study  Randomized study  Retrospective study  Prospective pilot-study | AFG from inner thigh for Breast deformity in Poland syndrome  AFG from thigh region+ BoNT-A for bilateral augmentation of the breast  Cell-assisted AFG from thigh or abdomen for Micromastia  Lipoaspirates from anterior and inner thigh with NAC for breast asymmetry | AFG + BoNT-A (1),  AFG w/o BoNT-A  AFG + BoNT-A  (1) vs. AFG with saline (2)  CAL (1) vs. standard AFG (2)  AFG harvested via NAC-enriched tumescent fluid (1) vs. standard AFG (2) | 20 (f),  16-45 y,  mean BMI 19.38 (1), 21.02 (2)  15 (f)  34 (f), 18-45 y, BMI 18-25  15 (f), 23-29 y, mean BMI 22.4 | 3, 6 m  3,6 m  6 m  6 m | Harvesting / Processing via Coleman technique, adding of BoNT-A (1) after centrifugation, injection into breast area  Coleman protocol, mixing with BoNT-A (1) or saline (2) after centrifugation of fat  Liposuction, centrifugation, isolation of SVF and addition to AFG (1), injection according to Coleman  Liposuction with Klein solution with (1) or w/o (2) NAC, sedimentation and decantation, injection | Fat retention rate 3 and 6 m post-op (3D imaging), Breast-Q, number of surgeries to achieve 80% Fat retention  AFG volume (3D imaging, patient and doctor assessment)  QLQ-30, fat retention rate (MRI), breast uplift value  Breast volumetric analysis | Higher retention rate in (1)*  3 surgeries to achieve result in both groups (median)  Higher satisfaction with breast in (1)*  Higher retention rate in (1) *  Comparable complication rate  Higher uplift value in (1) *  Higher fat retention in (1)  Similar QoL  Lower resorption rate in (1)  No post-op complication after 6 m |
| Ex-vivo-expanded autologous adipose tissue-derived stromal cells ensure enhanced fat graft retention in breast augmentation: A randomized controlled clinical trial. (Kolle *et al.* 2021) | Blinded  RCT | ASC-enriched AFG from abdomen/thigh/hips for cosmetic breast augmentation | AFG with ex-vivo expanded ASC (1) vs. non-enriched AFG (2) | 16 (f), 18-50y, BMI 18-30 | 18 m | Harvest, isolation and washing of ASC, mixing of AFG with ASC, injection | Volume retention (MRI + photographs assessed by blinded plastic surgeons) | Higher retention rate in (1)*  Superior objective results in photographs in (1)* |
| A Prospective Study of the Efficacy of Cell-Assisted Lipotransfer with Stromal Vascular Fraction to Correct Contour Deformities of the Autologous Reconstructed Breast. (Jeon *et al.,* 2021) | Prospective study | CAL with and without SVF for breast asymmetry after total mastectomy and autologous reconstruction | AFG with SVF (1)  Vs. AFG w/o SVF (2) | 20 (f), mean age 46.6 y, mean BMI 23.4 | 12 m | Liposuction from abdomen or flank, double centrifugation, isolation of SVF, transplantation or naïve AFG or CAL s.c. into breast tissue | Variance of breast volume  Patient satisfaction  Complications | Higher survival rate of AFG with (1)  Improved patient satisfaction in (1) |
| Cell-Assisted Lipotransfer for Cosmetic Breast Augmentation: Supportive Use of Adipose-Derived Stem/Stromal Cells  (Yoshimura *et al*., 2020) | Prospective Study | CAL for cosmetic breast augmentation, harvest from abdomen, thigh, lower legs | CAL  (SVF either mixed into AFG or  resuspended in saline and injected into breast mounds after lipoinjection) | 40 (f), 20-49 y, mean BMI 19.1 | 2 m | Harvesting of AFG, washing or centrifugation isolation and processing of SVF, enrichment of grafted fat with SVF or direct injection of diffused SVF into breast tissue after AFG , injection of circa 270 mL AFG into breast tissue | Breast volume  Postoperative atrophy, post-op cyst formation, patient satisfaction | Volume increase by 100 to 200 mL, minimal postoperative atrophy, cyst formation / microcalcification in 4 patients, high patient satisfaction |
| Effect of Salvia Miltiorrhiza Injection in Patients With Autologous Fat Grafting to the Breast: A Preliminary Comparative Study.  (Zheng *et al*., 2019) | Preliminary comparative study | Intravenous Salvia Miliorrhiza (SM) infusions for breast augmentation with AFG from thigh or abdomen | AFG + SM (1) vs. AFG alone (2) | 15 (f), 10 patients in (1), 5 patients in (2), mean age 30.4 y, mean BMI 21.6 | 4 w | Tumescent liposuction,s.c. injection i.v. SM from 1 w pre-op until 4 w post-op (1) | Breast volume after AFG (3D scan), breast morphology, cyst formation | improvement in breast volume after AFG in (1) and (2)  good breast morphology and cyst absence in (1) |
| Does Stromal Vascular Fraction Ensure a Higher Survival in Autologous Fat Grafting for Breast Augmentation? A Volumetric Study Using 3-Dimensional Laser Scanning.  (Chiu *et al*., 2019) | Retrospective Study | SVF enriched AFG from abdomen, flanks, hips, thighs, calves for breast augmentation | AFG with SVF (1)  Vs. traditional AFG (2) | 206 (f), mean age 37 (1) and 33 (2), mean BMI 20.3 (1) and 18.8 (2) | 3, 6,12 m | UAL, centrifugation and isolation of SVF (1) and transplantation of SVF into AFG (1), injection (amount individualized) | Survival rate of transplanted fat (3D scanning)  Postoperative Complications | Lower fat survival rate in (1)*  Higher complication rate in (1)* |
| Enrichment of autologous fat grafts with ex-vivo expanded adipose tissue-derived stem cells for graft survival: a randomized placebo-controlled trial. (Trojahn Kolle *et al*., 2013) | Triple blind, placebo-controlled RCT | Ex-vivo expanded ADSCs to enrich lipoaspirate from abdomen | AFG with ADSCs (1) vs. AFG w/o ADSCs (2) | 10 patients, 18-45 y, BMI 20-30 | Up to 5 m | Liposuction via Coleman technique, enrichment with ex-vivo ADSCs in (1), s.c. injection as bolus of (1) and (2) on different locations on the upper arm | Residual graft volume | higher residual volume in (1)*  no serious adverse events in either group |
| Breast Reconstruction with Autologous Fat Graft Mixed with Platelet-Rich Plasma.  (Gentile *et al.,* 2013) | Prospective Study | AFG + PRP to improve soft tissue defects | AFG + PRP (1) vs. AFG alone (2) | 100 (f) 19-60 y | 1 y | Fat grafting via Coleman technique (1) and (2), mixing of AFG and PRP in (1) | Contour restoring, residual volume (3D) after 1 y | 69% contour maintenance and 3D volume in (1)  39% contour maintenance and 3D volume in (2) |
| A Comparative Translational Study: The Combined Use of Enhanced Stromal Vascular Fraction and Platelet-Rich Plasma Improves Fat Grafting Maintenance in Breast Reconstruction.  (Gentile *et al.* 2012) | Comparative study | AFG enhanced with SVF and PRP for breast soft tissue defects | AFG + SVF (1) vs.  AFG+ PRP (2)  vs. Control (3) | 33, 13 (1), 10 (2), 10 (3), 19-60 y | 1 y | Washing and centrifugation of fat, addition of SVF (1), centrifugation of purified fat, mixed with PRP (2), centrifuged lipoaspirate processed via Coleman technique (3) | Maintenance of contour restoring and volume (3D) | 63 % maintenance in (1)  69 % maintenance in (2)  39 % maintenance in (3)  Improvement with e-SVF or PRP |

Abbreviations: N: Number of patients included, AFG: Autologous fat grafting, BoNT-A: Botulinum Toxin Type A, w/o: Without, f:female, m:male, y:years, w:weeks, BMI: Body Mass Index, m:months, Breast-Q: Breast-Questionnaire, RCT:Randomized Controlled Trial, PWAS:Poloxamer wash absorption system, NSM:Nipple sparing mastectomy, n.a.:not applicable, CAL:Cell-assisted Lipografting, SVF:Stromal vascular fraction, ADSC: Adipose-derives stem cells, QLQ-30: Quality of Life score, MRI: Magnet resonance imaging, QoL:Quality of Life, NAC: N-Acetylcysteine, UAL:Ultrasound-assisted Liposuction, SM: Salvia Miliorrhiza, PRP:Platelet-rich Plasma
