# Supplementary material for: Optimizing Autologous Fat Grafting: A Systematic Review of Enhancement Strategies and Graft Survival
Source: Aesthet Surg J. 2025 Nov 19;46(11):1246–52. doi: 10.1093/asj/sjaf242 (PMC13668368; doi:10.1093/asj/sjaf242)
Supplement: Supplementary file 1 [file sjaf242_supplementary_data.zip › Supplemental Table 3.docx]

**Supplemental Table 3**. Comparative Overview of Investigated Enhancement Strategies for Autologous Fat Grafting

| Enhancement strategy | Proposed Machanism of Action | Main Strengths | Limitations | Clinical Feasibility |
| --- | --- | --- | --- | --- |
| Adipose- Derived Stem Cells (ADSC)  Stromal Vascular Fraction (SVF)  Platelet-Rich-Plasma (PRP)  Botulinum Toxin Type A (BonT-A)  Vitamin D3 /  Calcitriol  N-Acetylcysteine (NAC)  Salvia miltiorrhiza  (SM) | Promote angiogenesis, differentiation into  adipocytes, and paracrine secretion of growth factors [6], [8], [9], [31]  Provides a heterogeneous mix of regenerative and endothelial cells enhancing neovascularization [8], [9], [31]  Delivers growth factors (VEGF, PDGF, TGF-β) promoting angiogenesis and graft survival [22], [26], [37], [39]  Reduces muscle contraction, improves  perfusion and angiogenic signaling [27], [28]  Regulates adipocyte differentiation and reduces inflammatory response [4], [11], [35], [36]  Antioxidant; protects adipocytes from oxidative stress during transplantation [29]  Increases VEGF expression and angiogenesis [17], [30] | Improved graft retention and vascularization; regenerative potential  Autologous, rich in regenerative cells, immediate preparation possible  Simple, autologous,  cost-effective; enhances  early graft take  Enhances retention; low  complication rate  Enhances adipocyte survival and vascularization  Reduces fat resorption and  inflammation  Promotes vascularization; low adverse effects | High cost, time-intensive cell isolation, regulatory restrictions [31], [32]  Variability in cell composition; limited standardization; ethical/regulatory barriers [31], [34]  Inconsistent preparation  protocols; short biological  activity [22], [39]  Mechanism not fully elucidated; limited data [27], [28]  Few studies; unclear optimal dosage and delivery method [4], [11]  Limited clinical data; unconfirmed long-term outcomes [29]  Only single preliminary studies; unclear dosing regimen [30] | Moderate – feasible in  specialized centers only  Moderate – requires  specialized lab facilities  High – easily applicable  in routine practice  High – readily implementable in standard clinical settings  Low to Moderate –  experimental stage  Moderate – potential for simple clinical integration  Low – experimental /  adjunctive use only |

Abbreviations: ADSC: Adipose-derived stem cells ; BoNT-A: Botulinum Toxin Type A; SVF:Stromal vascular fraction; NAC: N-Acetylcysteine; PDGF: Platelet-derived growth factor; PRP:Platelet-rich Plasma; SM: Salvia Miliorrhiza; TGF-β: Transforming growth factor-beta; VEGF: Vascular endothelial growth factor.
